# Supplementary material for: Dietary lipids shape cytokine and leptin profiles in obesity-metabolic syndrome implications: A cross-sectional study
Source: PLoS One. 2024 Dec 19;19(12):e0315711. doi: 10.1371/journal.pone.0315711 (PMC11658627; doi:10.1371/journal.pone.0315711)
Supplement: S1 Table — Analysis of interactions between inflammatory and hormonal biomarkers stratified by high versus low sugar intake in the obese group (n = 199). (DOCX) [file pone.0315711.s001.docx]

**S1 Table.** **Interaction between blood inflammation and hormonal markers in high–low sugar contents in the obese group**

| **Variables** | **Obese (n=199)** | | | | | | | |
| --- | --- | --- | --- | --- | --- | --- | --- | --- |
|  | **Low sugar vs High sugar** | | | | | | | |
|  | **OR** | **95% CI** | | ***p* *value*** | **OR*** | **95% CI** | | ***p* *value*** |
| Blood Inflammation markers | | | | | | | | |
| TNF-alpha (pg/mL) | | | | | | | | |
| < 29 | Reference | |  |  | Reference | |  |  |
| > 29 | 0.63 | (0.27 - | 1.50) | 0.305 | 0.59 | (0.24 - | 1.42) | 0.245 |
| Interlukin-6 (pg/mL) | | | | | | | | |
| < 30 | Reference | |  |  | Reference | |  |  |
| > 30 | 1.03 | (0.42 - | 2.51) | 0.941 | 0.93 | (0.37- | 2.33) | 0.892 |
| Myeloperoxidase (ng/mL) | | | | | | | | |
| < 87.8 | Reference | |  |  | Reference | |  |  |
| > 87.8 | 0.76 | (0.28- | 2.07) | 0.602 | 0.59 | (0.20- | 1.72) | 0.335 |
| Leptin (ng/mL) | | | | | | | | |
| < 5 | Reference | |  |  | Reference | |  |  |
| > 5 | 1.1 | (0.20- | 5.89) | 0.906 | 2.12 | (0.31 - | 14.45) | 0.440 |
| Insulin (uIU/mL) | | | | | | | | |
| < 12 | Reference | |  |  | Reference | |  |  |
| > 12 | 0.74 | (0.28 - | 1.95) | 0.544 | 0.72 | (0.26- | 1.93) | 0.514 |

Unadjusted odds ratio (OR), *OR adjusted for gender, age, physical activity, and exercise

**p value* < 0.05 is considered statistically significant.
