# Supplementary material for: Dietary lipids shape cytokine and leptin profiles in obesity-metabolic syndrome implications: A cross-sectional study
Source: PLoS One. 2024 Dec 19;19(12):e0315711. doi: 10.1371/journal.pone.0315711 (PMC11658627; doi:10.1371/journal.pone.0315711)
Supplement: S2 Table — Analysis of interactions between inflammatory and hormonal biomarkers stratified by high versus low saturated fat intake in the obese group (n = 199). (DOCX) [file pone.0315711.s002.docx]

**S2 Table. Interaction between blood inflammation and hormonal markers in high–low saturated fat contents in the obese group**

| **Variables** | **Obese (n=199)** | | | | | | | |
| --- | --- | --- | --- | --- | --- | --- | --- | --- |
|  | **Low satfat vs High satfat** | | | | | | | |
|  | **OR** | **95% CI** | | ***p* *value*** | **OR*** | **95% CI** | | ***p value*** |
| Blood Inflammation markers | | | | | | | | |
| TNF-alpha (pg/mL) | | | | | | | | |
| < 29 | Reference | |  |  | Reference | |  |  |
| > 29 | 0.7 | (0.28 - | 1.71) | 0.873 | 0.62 | (0.085 - | 2.81) | 0.988 |
| Interlukin-6 (pg/mL) | | | | | | | | |
| < 30 | Reference | |  |  | Reference | |  |  |
| > 30 | 1.78 | (0.92- | 3.47) | 0.09 | 2.03 | (1.00- | 4.11) | **0.047*** |
| Myeloperoxidase (ng/mL) | | | | | | | | |
| < 87.8 | Reference | |  |  | Reference | |  |  |
| > 87.8 | 1.93 | (0.56 - | 6.62) | 0.294 | 1.72 | (0.48 - | 6.13) | 0.401 |
| Leptin (ng/mL) | | | | | | | | |
| < 5 | Reference | |  |  | Reference | |  |  |
| > 5 | 1.35 | (0.69 - | 2.63) | 0.372 | 1.65 | (0.82 - | 3.31) | 0.154 |
| Insulin (uIU/mL) | | | | | | | | |
| < 12 | Reference | |  |  | Reference | |  |  |
| > 12 | 1.20 | (0.60 - | 2.40) | 0.587 | 1.33 | (0.65- | 2.72) | 0.422 |

Unadjusted OR, *OR adjusted for gender, age, physical activity, and exercise

**p value* < 0.05 is considered statistically significant.
