# Supplementary material for: Dietary lipids shape cytokine and leptin profiles in obesity-metabolic syndrome implications: A cross-sectional study
Source: PLoS One. 2024 Dec 19;19(12):e0315711. doi: 10.1371/journal.pone.0315711 (PMC11658627; doi:10.1371/journal.pone.0315711)
Supplement: S3 Table — Analysis of interactions between inflammatory and hormonal biomarkers stratified by high versus low total fat intake in the obese group (n = 199). (DOCX) [file pone.0315711.s003.docx]

**S3 Table.** I**nteraction between blood inflammation and hormonal markers in high–low total fat contents in the obese group**

| **Variables** | **Obese (n=199)** | | | | | | | |
| --- | --- | --- | --- | --- | --- | --- | --- | --- |
|  | **Low fat vs High fat** | | | | | | | |
|  | **OR** | **95% CI** | | ***p value*** | **OR*** | **95% CI** | | ***p value*** |
| Blood Inflammation markers | | | | | | | | |
| TNF-alpha (pg/mL) | | | | | | | | |
| < 29 | Reference | |  |  | Reference | |  |  |
| > 29 | 0.77 | (0.12 - | 4.75) | 0.783 | 0.63 | (0.09 - | 4.06) | 0.630 |
| Interlukin-6 (pg/mL) | | | | | | | | |
| < 30 | Reference | |  |  | Reference | |  |  |
| > 30 | 1.72 | (0.91 - | 3.24) | 0.09 | 1.84 | (0.95- | 3.55) | 0.068 |
| Myeloperoxidase (ng/mL) | | | | | | | | |
| < 87.8 | Reference | |  |  | Reference | |  |  |
| > 87.8 | 1.43 | (0.42- | 4.89) | 0.562 | 1.32 | (0.38- | 4.58) | 0.660 |
| Leptin (ng/mL) | | | | | | | | |
| < 5 | Reference | |  |  | Reference | |  |  |
| > 5 | 1.87 | (1.00 - | 3.50) | **0.048*** | 2.14 | (1.12 - | 4.10) | **0.021*** |
| Insulin (uIU/mL) | | | | | | | | |
| < 12 | Reference | |  |  | Reference | |  |  |
| > 12 | 1.64 | (0.86 - | 3.13) | 0.133 | 1.74 | (0.90- | 3.38) | 0.098 |

Unadjusted OR, *OR adjusted for gender, age, physical activity, and exercise

**p value* < 0.05 is considered statistically significant.
