# Supplementary material for: Dietary lipids shape cytokine and leptin profiles in obesity-metabolic syndrome implications: A cross-sectional study
Source: PLoS One. 2024 Dec 19;19(12):e0315711. doi: 10.1371/journal.pone.0315711 (PMC11658627; doi:10.1371/journal.pone.0315711)
Supplement: Attachment — Submitted filename: Point by point 24 10 24.docx [file pone.0315711.s005.docx]

'Point-by-point response to editors and reviewers'

According to comments, we agreed and corrected with all the comments raised by the editors and reviewers. We would like to take this opportunity to express our sincere thanks to the editors and reviewers who identified areas of our manuscript that needed corrections or modification. We would like also to thank you for allowing us to resubmit a revised copy of the manuscript.

| **Comments** | **Author’s Response** |
| --- | --- |
| **Reviewer 1** |  |
| This is an interesting study on “Dietary lipids shape cytokine and leptin profiles in obesity-metabolic syndrome implications: a cross-sectional study”. It’s an honour to have the opportunity to review the manuscript.  My comments are appended.  **Abstract:**  Abstract is written properly; however, I think it is better to elaborate the methodology of anthropometric and biochemical measurements. | Thank you for your valuable feedback. We included additional details about the procedures, tools, and protocols used for these measurements to enhance the reader's understanding.  See Abstract part |
| **Introduction:**  Introduction has been written in right direction. However, the author didn’t mention the context of the area and people’s ethnicity. It is okay, if this is the first ever study to explore this in the context of people all over the world, though it is worthy to mention. It is necessary to ensure external validity of any findings. | We acknowledge the importance of mentioning the specific demographic and geographic context to enhance the external validity of the findings. We provided details about the study population’s ethnicity and the regional context.  “While extensive research has been conducted globally on the relationship between diet, lipid metabolism, and obesity, there is a lack of studies that explore these factors within specific ethnic and geographical contexts. The social, cultural, and environmental factors unique to particular populations can significantly influence dietary patterns and their metabolic effects (24).Understanding these contextual variables is essential to ensuring the external validity of any findings and to allow for the generalization of results across diverse populations.”  See in the introduction part line 104-110 |
| **Methods:**  It is not clear how the sample size was calculated.  Is the semi-FFQ previously validated in Thailand? | We thank the reviewer for pointing out the need for clarification regarding the sample size calculation. We added sample size calculation into the statistical analysis part.  “The sample size calculation was conducted through two approaches to ensure adequate statistical power. First, for detecting genetic associations, a power analysis was performed with parameters of moderate effect size (r = 0.3), power (1-β) = 0.80, and significance level (α) = 0.05 for correlation analysis, indicating a minimum required sample of 374 participants. Second, for population proportion estimation, we used the formula n = [Z²p(1-p)]/d², where Z = 1.96 (95% confidence level), p = 0.424 (42.4% obesity prevalence in Thai population, 2021), and d = 0.05 (5% margin of error), which also yielded a minimum sample size of 374. To account for potential 10% data loss, we aimed to recruit 412 participants. The final study included 384 participants, exceeding the minimum required sample size of 374, thus ensuring adequate statistical power for both genetic association analysis and population proportion estimation”  See line of 195-205  The semi-FFQ was adapted from a version validated in Thailand for people with metabolic syndrome risk (Nirdnoy et al., 2023). While we made minor modifications to include additional local foods, the core structure remained consistent with the validated version and was reviewed by Thai nutrition experts. This limitation has been acknowledged in our discussion section.  “Although our semi-FFQ was adapted from a previously validated version for Thai adults with metabolic syndrome risk (Nirdnoy et al., 2023), and underwent expert review for our modifications of additional local foods, the modified version has not yet been specifically validated in our study context, which could affect the precision of dietary intake assessments.”  See Line 378-381 |
| **Results:** The results are written in good order.  **Discussion:** Discussion is written properly | Really thank you for your valuable and positive feedbacks. |
| **Reviewer 2** |  |
| The manuscript evaluates the relationship between inflammatory markers, hormones, and dietary fat, linking them to obesity in an Asian population. While the study presents valuable insights, revisions are required to enhance its clarity.  **Methods**  Line 96: Was there a maximum BMI threshold used as an inclusion criterion? | We have maximum BMI ≤40 kg/m^2^ (For Obese class III) and added into the inclusion criteria  “body mass index (BMI) ≥ 18.5 kg/m^2^ to 40 kg/m^2”^  Line 123 |
| Lines 105-107: Please, refine the language. The sentence is somewhat confusing. The same for lines 111-112. | We checked and refined the language in these sections. |
| Line 117: what is BIA? | We provided a clear explanation of BIA, or Bioelectrical Impedance Analysis in the text.  Line 146 |
| Line 138: Is there a reference used for the calculation of HOMA-IR? | Thank you for your question regarding the calculation of HOMA-IR. We added reference in to the methods  See line 167 |
| Line 142: standardize the citation of reagents/equipment | Thank you for your observation regarding the citation of reagents/equipment. We reviewed and standardized the citations of all reagents and equipment. Each reagent and piece of equipment is cited consistently, following the appropriate format  See line 178 |
| Line 143: explain the meaning of high-risk values: high risk for metabolic diseases? | High-risk values in this study refer specifically to biomarker thresholds associated with increased risk of both metabolic diseases and inflammatory complications  See line 172-178 |
| Line 149: What time frame was used to assess dietary intake? Even though the FFQ has already been published, it would be helpful to describe it briefly in this manuscript. | The time frame used to assess dietary intake was designed to assess dietary intake over a 1-month period. We briefly described FFQ as suggest.  See in the 184-188 |
| **Results**  Line 166: review the writing. | We rewrite the sentence  See line 221-222 |
| Line 167: Be specific in the text considering the comparison of obesity between genders, for example, showing the P-value. | We revised that “In the obese group, all anthropometric parameters after adjusted gender were significantly higher (*p* < 0.001), except for age and height.”  See line 231-234 |
| Line 175: It would be more effective to begin this section with the final sentence of the paragraph. | Corrected as suggest  See line 230-232 |
| Line 464 (table 2): Were the data adjusted for gender? This is important, as men and women exhibit considerable differences in body composition. I suggest showing both analyses. | Thank you for your importance of considering gender differences, especially in terms of body composition.  We updated the manuscript to reflect both sets of results. The findings for gender adjust analyses are presented in Table 2 |
| Line 195: According to figure 1B, HDL level is higher in the non-obese group. Please revise the text to reflect this information accurately. | We revised the text to accurately reflect the observation from Figure 1B, indicating that HDL levels are indeed higher in the non-obese group. The updated text can be found in the revised manuscript  See line 248-249 |
| Figure 1A: Create a graph with the y-axis divided into two segments to highlight the smaller values. | Thank you for your suggestion, we created a new graph as suggest.  See in figure 1 |
| Figure 1A-B: Some of the bars in the graphs appear to be missing SD. Improve the quality of the graphs. There are 2 legends in figure 1. | We fixed graph as suggest.  See in figure 1 |
| Lines 198-205: these data are presented in table 5 | Thank you for your observation. We confirm that these data are indeed presented in Table 4. We have clarified the text explained in table 5.  See line 252-264 |
| Lines 206-208: these data are presented in table 4 | We have clarified the the text explained in table 4.  See line 252-2257 |
| Line 202: the reported r value is associated with correlations involving TNF. Specify or provide the corresponding correlation values for leptin in the text. | We apologize for any confusion. We have now added the corresponding r values for leptin in the revised manuscript.    See line 259-263 |
| Table 5: the parameters in the columns (e.g., BL glucose) are somewhat unclear. It would also be more appropriate to include the abbreviations below each table for clarity. | We revised Table 5 to clarify as suggest.  See Table 5 |
| Lines 209-217: starting this topic with the final sentence of the paragraph may make it easier for readers to follow the data. | We revised the paragraph as suggest.  See line 252-254 |
| Line 212: refer to supplementary table 2 | Corrected as suggest  See line 273 |
| Line 213: refer to supplementary table 3 | Corrected as suggest  See line 266 |
| Line 215: the data are presented in table 4, not 5 (leptin versus sugar intake). | We checked and revised the paragraph to refer to Supplementary Table 1 instead of Table 5.  See line 273 |
| Discussion  Line 223: I suggest the authors discuss the higher prevalence of obesity observed among women, considering global data or specific to Asia. | We added a discussion considered relevant global data as well as data specific to Asia to provide a more comprehensive analysis.  See line 280-293 |
| What is the meaning of negative correlations of leptin, IL-6, TNF, and insulin with HDL? | Our negative correlations between HDL and inflammatory markers (TNF-α, IL-6), leptin, and insulin also suggest a relationship where inflammation may impair HDL synthesis and increase its catabolism. This could create a cycle where reduced HDL levels further compromise anti-inflammatory protection in obesity.  See line 302-305 |
| Line 254: reference? | Thank you for your feedback. We have now included a relevant reference  See line 358 |
| **Reviewer 3** |  |
| This study entitled Dietary lipids shape cytokine and leptin profiles in obesity-metabolic syndrome implications: a cross-sectional study is interesting. However, I have some comments.  1. "Obesity is a significant risk factor for metabolic syndrome, a cluster of conditions including diabetes, cardiovascular diseases (CVDs), dyslipidemia, and insulin resistance." There isn’t any references for this sentence. Please add reference like these studies: "Association of circulating adipokines with metabolic dyslipidemia in obese versus non-obese individuals" | We appreciate the reviewer’s insightful comment. We added the references as suggested  See in line 47-59 |
| 2. Its better to add some reports about the prevalence rate of obesity across the world based on some meta-analysis or epidemiological studies. | We added the references as suggested  See in line 47-59 |
| 3. The section does a good job of summarizing existing knowledge about obesity and inflammation, mentioning key inflammatory cytokines (TNF-α, IL-6) and leptin. However, it could delve a bit deeper into why these particular markers were chosen for the study. For instance, more emphasis on leptin's role in obesity-related complications could add depth. | We provided a more detailed rationale for choosing TNF-α, IL-6, and leptin as key markers of inflammation in the context of obesity.  See line 66-70 |
| 4. "Complex factors of genetic susceptibility, sedentary lifestyle, and high-calorie diet consumption comprise the etiology of obesity and metabolic syndrome, resulting in chronic energy imbalance and excessive body fat accumulation" please add references such as 10.1016/j.clinthera.2020.12.021 and 10.1002/ptr.7081 | We added the reference into the paragraph  See line 57 |
| 5. How did you calculate sample size for this study, it should be added to the method section. | This information has now been included in the statistics section of the manuscript  See in line 195-201 |
| 6. cutoff values for high-risk levels of TNF-α, IL-6, and leptin are provided, which is a strength. However, a brief explanation of why these specific thresholds were chosen (e.g., based on previous research or clinical relevance) would be useful. | We have specified that these thresholds were based on previously published studies demonstrating their clinical relevance in predicting inflammation-related complications such as insulin resistance and cardiovascular disease  See in line 171-178 |
| 7. Statistical Analysis: its better to explain the confounding or covariate used in this study. | We provided a detailed explanation of the confounders and covariates used in the statistical analysis. Specifically, age, gender, physical activity, and exercise were included as confounding variables, as these factors are known to influence the outcomes related to obesity and metabolic syndrome.  See line 211-213 |
| 8. Were any potential confounders (e.g., age, gender, physical activity) controlled for in the analysis, and how were they selected? | Age, gender, and physical activity were controlled for as confounders based on their established influence on both dietary lipids (exposure) and inflammatory markers (outcomes). These variables were included in our multivariable regression models to minimize bias.  See line 209-215 |
| 9. the statistical significance of these correlations (e.g., for sugar and leptin levels) should be interpreted more carefully. Although the correlations are statistically significant, are they clinically meaningful? The strength of the correlations (e.g., r-values) could be discussed to provide more context. | We have addressed this concern in our discussion, acknowledging that while the correlation between sugar and leptin is statistically significant (r = 0.110, p < 0.05), its weak strength limits its clinical relevance. We support this interpretation with evidence from previous studies that emphasize the distinction between statistical and clinical significance in metabolic research.  Sinha MK, Tetrault J, Ohannesian JP, et al. Serum immunoreactive leptin concentrations in normal-weight and obese humans. N Engl J Med. 1996;334(5):292-5. doi:10.1056/NEJM199602013340501  Gruzdeva O, Borodkina D, Uchasova E, Dyleva Y, Barbarash O. Leptin resistance: underlying mechanisms and diagnosis. Diabetes Metab Syndr Obes. 2019;12:191-8.  See line 252-264 |
| 10. In the discussion, its better to explain more about mechanism related to observed results. | We expanded the discussion to include the relevant biological mechanisms that contribute to the findings of our study.  See line 279-292 305-317, 329-352 |
